# Efficacy and Growth Performance between Two Different Ionophore Coccidiostats (Narasin and Salinomycin) in Broiler Chickens after Challenge with *Eimeria* spp.

**DOI:** 10.3390/ani14182750

**Published:** 2024-09-23

**Authors:** Monika Rogala-Hnatowska, George Gould, Shubhi Mehrotra, Aleksandra Drażbo, Paweł Konieczka, Prakash Ramasami, Krzysztof Kozłowski

**Affiliations:** 1 Elanco Poland Sp. z o.o., 00-843 Warszawa, Poland; monika.rogala-hnatowska@elancoah.com; 2 Elanco Animal Health, Hook RG27 9XA, UK; george.gould@elancoah.com; 3 Elanco Innovation and Alliance Centre, Bangalore 560017, India; shubhi.mehrotra@elancoah.com (S.M.); prakash.ramasami@elancoah.com (P.R.); 4 Department of Poultry Science and Apiculture, University of Warmia and Mazury in Olsztyn, Oczapowskiego 5, 10-719 Olsztyn, Poland; pawel.konieczka@uwm.edu.pl (P.K.); kristof@uwm.edu.pl (K.K.)

**Keywords:** broiler chickens, ionophore, anticoccidials, coccidiostats, efficacy, broiler performance, narasin, salinomycin

## Abstract

**Simple Summary:**

There has been an exponential increase in demand for poultry meat and eggs worldwide. Therefore, it is essential to keep coccidiosis under control in broilers to fulfill the increase in demand for protein. The objective of this study was to assess the efficacy of two ionophore coccidiostats against coccidiosis and their impact on broiler gut health and performance. Both the ionophores were effective in treatment against coccidiosis, and out of the two, narasin demonstrated superiority in terms of improved performance parameters compared with salinomycin. This finding is highly important as it significantly focuses on sustainable poultry and, in turn, can help prevent economic losses and maintain broiler health.

**Abstract:**

The objective of this study was primarily to assess the different performance impacts of two ionophore coccidiostats (narasin and salinomycin) used to manage coccidiosis. While both products may be efficacious in controlling disease challenges, previous literature has suggested that some ionophores are less well tolerated by the broiler chickens. In this study, we were particularly interested to know how the use of different coccidiostat programs translates into broiler health and performance, as measured by zootechnical parameters such as the feed conversion ratio, average daily gain, and final body weight. A total of 352 male Ross 308 one-day-old broilers were randomly divided into two treatment groups (T1 and T2). Treatment 1 included a basal diet (BD) + nicarbazin/narasin (Maxiban^®^, Elanco) at 100 ppm 0–24 days, narasin at 70 ppm 25–42 days, and (2) Treatment 2 included basal diet + nicarbazin/narasin at 100 ppm 0–24 days, salinomycin (Sacox^®^, Huvepharma) at 70 ppm 25–42 days. Efficacy and performance parameters, slaughter analysis, dry matter (DM) in litter, and intestinal integrity (I^2^) were measured for the broilers from both treatment groups. The findings demonstrated more favorable results for broilers reared in the group diet fed with narasin (in the finisher phase), including higher daily body weight gain, higher final body weight, lower feed conversion ratio value (improved feed efficiency), and higher European Production Efficiency Factor value, compared with the salinomycin-supplemented group.

## 1. Introduction

Avian coccidiosis caused by obligate intracellular parasites of the genus *Eimeria* is perhaps the most prevalent disease in the poultry industry, resulting in great financial pressures and economic losses as high as USD 3–4 billion per year due to impaired feed conversion rate, morbidity, intestinal lesions, poor weight gain, and, in some cases, mortality [1,2,3,4,5,6]. *Eimeria* spp. are highly adaptable and can survive in the same environmental conditions as the host poultry species [7]. 

Seven species of *Eimeria*, namely, *E. acervulina*, *E. brunetti*, *E. maxima*, *E. mitis*, *E. necatrix*, *E. praecox*, and *E. tenella*, are associated with infections in chickens through colonization of the gastrointestinal tract [8]. *Eimeria acervulina*, *Eimeria necatrix*, *Eimeria maxima*, and *Eimeria tenella* are reported to be the most pathogenic species [9]. Coccidiosis leads to decreased average daily weight gain, greater feed conversion, lower growth/final body weight, and increased mortality in poultry [10]. The control of coccidiosis has been an important parameter in the growth and success of the worldwide poultry meat industry [11]. Between the years 1961 and 2020, there was an exponential increase in the demand for poultry meat (9–133 million tons) and eggs (15–93 million tons) [12]. Therefore, it is critical to keep coccidiosis under control in commercial poultry to fulfill the increasing demand for protein [13].

Also, the widespread historical use of synthetic compounds in combating coccidiosis has resulted in anticoccidial resistance. In recent decades, ionophore coccidiostats added as feed additives have offered an additional solution for stable control of coccidiosis [14].

Ionophores (IONs) are polyether ionophorous antiparasitics derived by fermentation (*Streptomyces* and other actinomycetes) or synthetic/chemicals derived by chemical (CHE) synthesis. The ionophore coccidiostats approved for use globally are monensin, lasalocid, narasin, salinomycin, and semduramicin. Synthetic chemicals include halofuginone, decoquinate, robenidine, zoalene, and nicarbazin [14,15,16]. Maxiban^®^ (classified as a CHE for the purpose of analysis) is a feed additive from Elanco (Elanco Animal Health, 2500 Innovation Way, Greenfield, IN, USA), which is a combination product of narasin (ionophore) and nicarbazin (synthetic compound) in a 1:1 ratio. Combining low levels of narasin and nicarbazin creates a synergistic effect that results in a dual mode of action, protecting birds against coccidiosis and subsequent enteritis [17,18]. 

Ionophore coccidiostats were first licensed over 50 years ago in 1971 and have been commercially used since then. Monensin was the first chemically characterized and commercialized ionophore [19,20]. While they remain highly effective, there have been several attempts to further improve their efficacy through combinations with other drugs [19].

The growth of the poultry industry today is mostly due to the extensive use of ionophore coccidiostats [20]. Narasin is a polyether coccidiostat (produced by fermentation) obtained from *Streptomyces aureofaciens* and a derivative of salinomycin with an additional methyl group [21]. Salinomycin, an ionophore coccidiostat, is another commonly used poultry feed supplement against *Eimeria* spp. [22]. 

The objective of this comparative study was to assess the efficacy and performance of two different potentiated monovalent ionophores, i.e., narasin and salinomycin, in a shuttle program with Maxiban^®^ in chickens. The birds received a coccidia challenge by using a 40× dose of a commercial anti-coccidial vaccine. Performance and health were evaluated to assess how the two coccidiostats differed in their management of this challenge.

## 2. Materials and Methods

### 2.1. Ethical and Study Approvals

All procedures in this study were evaluated and approved by the Local Animal Care and Ethics Committee in Olsztyn (UWM), Poland (Resolution No. 12/2022 from 16 March 2022), and were performed in accordance with the principles of the EU (recommendation 2007/526/CE) and the Polish Law on Animal Protection.

### 2.2. Experimental Design, Housing, and Management of Birds

A total of 352 male Ross 308 one-day-old broilers from a commercial hatchery were randomly divided into 2 treatment groups. Every treatment consisted of 16 pens (16 replicates) with 11 birds each—resulting in 176 birds per treatment. The two treatments were as follows: (1) Treatment 1—basal diet (BD) + nicarbazin/narasin (Maxiban^®^, Elanco) at 100 ppm for 0–24 days, narasin (Monteban^®^, Elanco) at 70 ppm for 25–42 days, and (2) Treatment 2—BD + nicarbazin/narasin (Maxiban^®^, Elanco) at 100 ppm for 0–24 days, salinomycin (Sacox^®^, Huvepharma) at 70 ppm 25–42 days. Both treatment groups were challenged with a 40-dose Evant vaccine (Hipra, Amer, Spain) provided by poultry veterinarians (Avipoint, Olsztyn, Poland), containing *Eimeria acervulina*, *Eimeria maxima*, *Eimeria praecox*, *Eimeria mitis*, and *Eimeria tenella* strains, into the crop on day 14 and 21 of the trial.

As stated in the study objectives, the primary goal of this study was to compare the direct impact of two of the most widely used coccidiostats in commercial poultry. Both narasin and salinomycin have been used to control coccidiosis in broiler production for many years, and many studies have demonstrated their efficacies in controlling this disease [23,24]. While one might assume that if both products are efficacious, then they are comparable, the literature suggests that some ionophore molecules have an effect on broiler feed intake and thus could impact broiler performance parameters [25,26]. This study primarily aimed to quantify any differences between narasin and salinomycin. This study was designed to maximize the value of any comparison; thus, we limited the treatment groups to enable a direct comparison and increase the replicates of each group to give greater power to the data generated.

The birds were kept in floor pens, 32 pens in total and 0.75 m^2^ each, with netting walls to avoid migration and pelleted straw as bedding material. Prior to the arrival of the birds, the pens were thoroughly cleaned and disinfected with a non-toxic solution. This was performed 7 days before the birds’ arrival on an “all in–all out” basis. The final body weight (BW) per pen was not higher than foreseen in EU regulations—39 kg/m^2^.

The trial was conducted in a poultry house with windows. The house was provided with artificial programmable lights and climate, automated electric heating, and forced ventilation. The heating and light program was in accordance with the recommendations of the Ross Broiler Management Manual [27]. The house was lit by programmable artificial light. This study followed a standard lighting program (per day) including 23 h of light and 1 h of dark (0–7 days of age) and 18 h of light and 6 h of dark (from day 7 to 3 days prior to slaughter). The total length of the study period was 42 days.

### 2.3. Basal Diet and Feed Additives

A two-phase feeding scheme was used (starter phase: 0–24 days and grower phase: 25–42 days), and feed and water were provided to the birds ad libitum. Wheat/corn/soya/rapeseed-based mash diets were provided to the broilers from feeders (one per pen; 15 cm × 30 cm). Basal feed mixtures (3 tons of starter and 6 tons of grower diet) were prepared by Agrocentrum Sp. z o.o. (Pisz, Poland) and delivered to the University of Warmia and Mazury in Olsztyn, Poland, where the experimental diets were prepared, and the feeding experiment was conducted. The composition and nutritional value of the basal diets are shown in Table 1. 

Basal diets were analyzed for crude protein, crude fiber, crude fat, dry matter, ash content (UWM Olsztyn, Poland), and coccidiostats content (Eurofins laboratory, Barcelona, Spain). The nutritional value of all diets corresponded to the nutrient requirements of Ross 308 broiler chickens [27].

### 2.4. Applied Experimental Challenges

The experimental treatment groups (T1 and T2) were challenged with a coccidia vaccine and an addition of coccidiostats to the diet (Table 2). The sources of coccidiostats used in the diets were the following commercial preparations: Maxiban in both groups Monteban^®^ by Elanco as a source of narasin (T1), and Sacox^®^ by Huvepharma as a source of salinomycin (T2). Both coccidiostats were used at a dose of 70 ppm. Maxiban was used at a dosage of 100 ppm.

The birds in the two treatment groups were challenged into crops with an Evant vaccine (40 times the recommended dose) on days 14 and 21 of the trial, according to Kozlowski et al. [28]. The vaccine was provided by poultry veterinarians (Avipoint, Olsztyn, Poland) and contained *Eimeria acervulina* (003), *Eimeria maxima* (013), *Eimeria mitis* (006), *Eimeria praecox* (007), and *Eimeria tenella* (004) strains of *Eimeria*.

The coccidia vaccine was administered directly into the crop with the use of a cannula. The degree of intestinal mucosa damage was evaluated by a poultry disease specialist based on any gross pathological changes on day 21 and day 28 of age, according to a previously described study using the health tracking system (HTSi) protocol established by Elanco Animal Health [29].

#### Health Tracking System (HTSi)

The health tracking system (HTSi) is an independently verified broiler benchmarking platform that provides a record of bird health based on necropsies, enabling producers to track bird performance and health prior to reaching processing. HTSi was developed by Elanco and has been in operation since 1995. The system was established to improve the understanding of flock health, support timely data-based decisions, and provide robust benchmarking with the overall goal of better performance, profitability, and animal welfare.

The I^2^ index is a weighted score, unique to HTSi, that analyzes the overall intestinal health of flocks. The index can function as a driver of broiler health and performance as a link has been established between better intestinal integrity and improved average daily gain (ADG), the feed conversion ratio (FCR), and the European production efficiency factor (EPEF) [30]. The score established in the I^2^ index is based on the evaluation of 23 key lesions, including coccidia, reviewed during necropsies. Data from the lesions tracked are used to calculate an I^2^ score, with a perfect intestinal integrity index score of 100 meaning no potential loss from gut health is detected.

### 2.5. Efficacy and Performance Parameters

The body weight of broilers (pen basis) was measured on days 1, 21, 35, and 42, whereas feed consumption and the feed conversion ratio were analyzed for the two treatment groups over experimental periods, i.e., 0–21, 22–35, 0–35, 36–42, and 0–42 days.

The average weight gain per bird, in each period (AWG), as calculated as:

AWG = F − S(1)

where F = average weight of the live birds in the pen on the day of weighing;

S = average weight of live birds in the pen at the first weighing.

The average feed consumed per bird, per day (ADFI) for the period, was calculated according to the following formula:

(2) $$\mathrm{ADFI}=\frac{A}{\left(B*C)+(D\right)}$$

A = Total feed consumed per pen for that period;

B = Number of surviving birds;

C = Day of study or the number of days for that period;

D = The sum of the days on which birds that died (+culled) were alive (in this study).

The feed conversion ratio for the period (FCR) was calculated as follows:

(3) $$\mathrm{FCR}=\frac{\mathrm{Feed}\;\mathrm{consumed}\;\mathrm{for}\;\mathrm{the}\;\mathrm{period}\;\mathrm{in}\;\mathrm{each}\;\mathrm{replicate}}{\begin{array}{l} \mathrm{Total}\;\mathrm{weight}\;\mathrm{gain}\;\mathrm{for}\;\mathrm{the}\;\mathrm{period}\\ \left(\mathrm{including}\;\mathrm{the}\;\mathrm{WG}\;\mathrm{from}\;\mathrm{dead}\;\mathrm{and}\;\mathrm{euthanized}\;\mathrm{birds}\right) \end{array}}$$

The European Production Efficiency Factor (EPEF) was calculated as:

(4) $$\mathrm{EPEF}=\frac{\mathrm{Liveweight}\;\left(\mathrm{kg}\right)\times \mathrm{Liveability}\left(\%\right)}{\mathrm{Age}\;\mathrm{at}\;\mathrm{depletion}\;\left(\mathrm{days}\right)\times \mathrm{Feed}\;\mathrm{conversion}\;\mathrm{ratio}\;\left(\mathrm{kg}/\mathrm{kg}\right)}\times 100$$

The results of slaughter analysis (day 42, one bird/pen with BW close to average BW of each treatment group) included carcass weight (CW), dressing percentage, breast meat yield, and bowel content (heart, gizzard, and liver).

Lesion scores were evaluated using method of Johnson and Reid [31] by randomly selecting one bird from each pen (16 birds per treatment) on days 21 and 28; 64 birds in total were necropsied. HTSi data were carried out for the birds on days 21 and 28. HTSi was developed by Elanco^TM^ and has been in operation since 1995. The system was established to improve the understanding of flock health, support timely data-based decisions, and provide robust benchmarking with the overall goal of better performance, profitability, and animal welfare.

Dry matter (DM) in the litter was measured at the end of this study by taking five samples of about 0.1 kg from 5 different points (4 corners and the center of the pen, excluding the areas directly under the heater and the drinker), which were mixed. Moisture was determined after being kept in a forced air oven at 75 °C for 48 h.

### 2.6. Disposition of Birds

All pens were assessed once daily with pen-side observations for general health. Employees handling birds in pens used face masks and gloves. Birds found dead were removed from the pen. Visibly sick birds were humanely euthanized and necropsied. The euthanasia method was according to the EU directive. Euthanasia was performed by trained personnel deemed proficient in the method. Birds that died or were removed from the experiment were weighed, and body weight and feeding period were considered in the calculation of growth performance indices.

### 2.7. Statistical Analysis

Comparisons of mean differences between the two groups for all analyzed means were performed by *t*-tests using SAS/STAT^®^ software (Version 9.4 of the SAS System for Windows, Copyright^©^ 2002−2012 by SAS Institute Inc., Cary, NC, USA). Significance was declared at *p* (probability) < 0.05, and 0.05 ≤ *p* < 0.10 was considered a near-significant trend.

## 3. Results

### 3.1. Feed Analysis

Both the starter and the grower basal meals, as well as experimental diets, were analyzed (Table 3 and Table 4). The analyzed values were found to be in line with the calculated values.

The litter in all the pens was of good quality. The dry matter (DM) in the litter was high, from 84.43% in T1 to 84.24% in T2 (day 42), and the mean difference was not statistically significant between the two treatment groups (*p* = 0.737).

### 3.2. Gut Health and Intestinal Integrity (I^2^)

On day 21, the chickens in the T1 group had a total intestinal integrity of 94.7, while the chickens in the T2 group had a total of 95.2. On day 28, the I^2^ was 95.4 for T1 and 95.0 for T2 birds. The overall HTSi results are presented in Table 5. The intestinal integrity in both groups was not significantly different. The other intestinal integrity components were measured, and the only significant difference was observed in the level of mucus content.

### 3.3. Growth Performance of Birds

The growth performance results are presented in Table 6.

At the start, the average body weight of day-old chickens was 39.3 g. During the experimental period on days 22–35, the birds from the T1 group gained significantly (*p* = 0.011) more weight than the T2 birds. Throughout the experimental phase (0–35 days), the birds in the T1 group were significantly heavier (*p* = 0.017) than the T2 birds. During the entire experiment (0–42 days), the T1 group birds were nearly significantly higher in BW and DWG (*p* = 0.077) in comparison with the T2 group birds. During the starter period (0–21 days), the results of feed intake (DFI) of both coccidiostat-supplemented groups (T1 and T2) were similar in values. During the experimental period on days 22–35, the birds in the T1 group consumed significantly more feed (*p* = 0.022) than the birds in the T2 group. During the experimental period on days 0–35, the birds in the T1 group consumed a higher amount of feed than the birds in the T2 group. For the entire experiment (0–42 days), the DFI calculated for the T1 birds was nearly significantly (*p* = 0.078) higher than for the T2 birds.

FCR was almost similar. Negligible differences were observed between the two treatments during the entire study, but the difference was not statistically confirmed.

The EPEF value calculated for the T1 birds was higher compared with the T2 birds; however, the difference was not statistically confirmed.

The liveability in the flock was 99.4% for both treatment groups throughout the experiment.

### 3.4. Slaughter Analysis

Representative birds from each treatment group were selected for slaughter analysis (Table 7). The results indicated that birds and carcasses from group T1 were near-significantly and significantly heavier than T2 birds (*p* = 0.061 and 0.046, respectively). The dressing percentage (DP) was nearly significantly (0.062) higher in the T1 group birds than in the T2 group birds. There were no significant differences in breast muscle or edible bowel (heart, gizzard, and liver) content between the two groups.

### 3.5. Lesion Scores

The lesion scores taken from the intestine of one randomly selected bird per pen are summarized in Table 8. No lesion scores were seen in birds from either group with *Eimeria tenella*. Fewer scattered lesions were seen in the birds from both groups with *Eimeria acervulina*. With *Eimeria maxima*, there were >five cases in the T1 group on day 21 and day 28.

## 4. Discussion

In this paper, we report the efficacy and performance of two different monovalent ionophores, i.e., narasin and salinomycin, in a shuttle program with Maxiban^®^ in chickens challenged by using a 40× the recommended dose of a commercial anti-coccidial vaccine. In addition, differences in performance and health were evaluated between the two coccidiostats in terms of impact on broiler gut health and performance.

*Eimeria* protozoa, which are responsible for causing parasitic infections such as coccidiosis, have major impacts on the morphology of the intestine, affecting digestion, micro-absorption of nutrients, and absorptive surface area [32,33,34,35]. The intestinal integrity in both groups was comparable, as the majority of factors were included in the composition of this index. Anti-protozoal protection, supported by the immunity of the birds in both groups, was probably maintained at similar levels. The vaccine challenge, in contrast to regular use, did not cause the clinical outbreak of coccidiosis, which indicates that the protective activity of the anticoccidials in both groups was maintained. The only statistically significant difference was observed in the level of excessive mucus in the intestines. In some cases, excessive mucus can be a good environment for anaerobic bacteria development, which may have a huge impact on the final performance of fast-growing broilers [36].

Growth performance is the most valuable index for monitoring poultry production [37]. The narasin-supplemented group (T1) showed improved performance parameters in terms of average body weight, daily feed intake, and EPEF. The improvement in growth parameters with narasin could have been due to its ability to restore the intestinal microbial balance, which then invigorated the secretion of endogenous digestive enzymes, improved the gut passage rate, and boosted intestinal morphology and nutrient absorption. Narasin may have also indirectly mitigated the inflammatory processes of coccidiosis by disrupting the replication of *Eimeria* [38,39]. It was also reported previously that narasin supplementation led to an improved feed conversion ratio in broilers [40]. A recent study by Abdelhady et al. reported that ionophores have the power to disrupt the ion gradients across the parasitic cell membrane [39].

However, the lower daily feed intake found in the salinomycin-supplemented broilers is in line with findings from previous studies that found decreased body weight, poor feed consumption, and a lower feed conversion ratio with therapeutic doses of salinomycin and even with higher doses of salinomycin in broilers [25,41,42]. Our study did not identify any significant differences in the lesion score between the two ionophores, but a previous study indicated that narasin supplementation led to a reduction in gizzard lesions in broilers [25,42,43]. In addition, previous studies have indicated that the use of narasin increasingly reduced intestinal damage in broilers, which is also the largest immune organ in broilers.

Additionally, these substances are highly efficacious against *Eimeria* and are, therefore, expected to reduce intestinal loads of parasites and secondary pathogenic bacteria, leading to a decrease in the corresponding host inflammatory responses. Under the conditions of the present study, both the examined ionophore coccidiostats were effective in treating coccidiosis and reinstating the measured parameters to optimum levels [39].

## 5. Conclusions

In our study, both coccidiostats were effective in treatment of coccidiosis and mitigation of the damage caused by the parasite, enabling desirable broiler performance. However, among the two coccidiostats, the results suggest that narasin (70 ppm) should be considered as a superior coccidiostat in comparison with salinomycin (70 ppm) in the finisher period (25–42 days) because of the enhanced performance parameters specifically in terms of average body weight, daily feed intake, intestinal integrity, and EPEF.

## Figures and Tables

**Table 1:** Calculated composition and nutritional value of basal diets (BDs)—starter and grower, % (as-fed basis).

Feed Composition	Starter (0−24 Days)	Grower (25−42 Days)
Wheat	45.00	55.00
Soybean meal	25.50	21.50
Corn	20.04	10.80
Rapeseed meal	3.00	5.00
Soya oil	2.74	4.63
NaCl	0.33	0.34
Limestone	1.26	1.17
Mono calcium phosphate	0.97	0.65
Methionine	0.32	0.23
L-Lysine	0.38	0.21
L-Threonine	0.08	0.09
Ronozyme^®^ P	0.01	0.01
Ronozyme^®^ WX	0.02	0.02
Choline chloride	0.10	0.10
Vit-Min-premix	0.25	0.25
ME, kcal/kg	2950	3050
Crude protein	21.00	20.00
Lysine	1.30	1.10
Methionine	0.62	0.52
Methionine + Cystine	1.00	0.90
Threonine	0.83	0.80
Calcium	0.85	0.75
Available phosphorus	0.33	0.27
Sodium	0.15	0.15

ME, metabolizable energy.

**Table 2:** Experimental treatments.

Treatment Group	Product and Coccidiostats	0–24 Days	25–42 Days
T1	Maxiban^®^/Monteban^®^	Maxiban^®^ 100 ppm	Monteban^®^ 70 ppm
T2	Maxiban^®^/Sacox^®^	Maxiban^®^ 100 ppm	Sacox^®^ 70 ppm

**Table 3:** Analysis of starter and grower basal diets, % (as fed basis).

Diet	DM	CP	EE	CA	CF
Starter (0−21 d)	89.9	21.10	5.58	4.64	3.26
Grower (22−42 d)	90.0	20.16	6.69	4.53	3.05

DM—dry matter; CP—crude protein; EE—ether extract; CA—crude ash; CF—crude fiber.

**Table 4:** Analysis of coccidiostat content in the experimental diets.

Treatment	Coccidiostat Content (mg/kg)
T1 (0−21 d) T1 (22−42 d)	Maxiban^®^ (narasin-51 and nicarbazin-43) Monteban^®^ (narasin-68)
T2 (0−21 d) T2 (22−42 d)	Maxiban^®^ (narasin-49 and nicarbazin-50) Sacox^®^ (salinomycin-74)

**Table 5:** Mean scores of intestinal integrity lesions in both treatment groups.

Intestinal Integrity Lesions	Treatment (Mean d21 and d28)		*p*-Value
	T1	T2	
I^2^	95.07	95.09	0.979
Cecal Foamy Material	0.19 ^y^	0.38 ^x^	0.098
Cellular Sloughing	0.09	0.13	0.694
Feed Passage	0.09	0.13	0.694
Hyperemia	0.00	0.03	0.321
Mucus Content	0.41 ^b^	0.66 ^a^	0.046
Thin Intestinal Walls	0.25 ^y^	0.47 ^x^	0.070
Watery Content	0.25	0.22	0.772

T1—Maxiban^®^/Monteban^®^; T2—Maxiban^®^/Sacox^®^. Values in the same row with no common superscript (a,b) are significantly different (*p* < 0.05), and 0.05 ≤ *p* < 0.10 (x,y) is considered a near-significant trend.

**Table 6:** Growth performance of broilers fed the experimental diets.

Specification	Treatment		SEM	*p*-Value
	T1	T2		
BW, g				
Day 1	0.039	0.039	0.001	0.212
Day 21	0.813	0.818	0.012	0.685
Day 35	2.174 ^a^	2.083 ^b^	0.252	0.017
Day 42	2.937 ^x^	2.858 ^y^	0.217	0.077
DWG, g				
Days 0−21	36.9	37.1	0.601	0.679
Days 0−35	61.0 ^a^	58.4 ^b^	7.220	0.017
Days 0−42	69.0 ^x^	67.1 ^y^	5.171	0.077
Days 22−35	97.2 ^a^	90.3 ^b^	19.189	0.011
Days 36−42	111.0	111.0	0.026	0.996
DFI, g				
Days 0−21	46.7	47.4	2.022	0.126
Days 0−35	94.5	92.7	4.967	0.127
Days 0−42	122.4 ^x^	119.4 ^y^	8.556	0.078
Days 22−35	148.5 ^a^	143.0 ^b^	15.715	0.022
Days 36−42	257.3	256.2	3.094	0.727
FCR, kg/kg				
Days 0−21	1.267	1.280	0.037	0.397
Days 0−35	1.454	1.461	0.019	0.588
Days 0−42	1.681	1.683	0.006	0.845
Days 22−35	1.581	1.595	0.039	0.605
Days 36−42	2.350	2.324	0.071	0.434
EPEF	410.1	402.0	22.073	0.271
Liveability, %	99.4	99.4	0.000	1.000

T1—Maxiban^®^/Monteban^®^; T2—Maxiban^®^/Sacox^®^; BW—body weight; DFI—daily feed intake; DWG—daily weight gain; FCR—feed conversion ratio; EPEF—European Production Efficiency Factor; SEM—standard error mean. Values in the same row with no common superscript (a,b) are significantly different (*p* < 0.05), and 0.05 ≤ *p* < 0.10 (x,y) is considered a near-significant trend.

**Table 7:** Slaughter analysis on day 42 of the trial.

Specification	Treatment *		SEM	*p*-Value
	T1	T2		
BWbs, kg	2.976 ^x^	2.881 ^y^	0.270	0.061
CW, kg	2.163 ^a^	2.083 ^b^	0.225	0.046
DP, %	72.7 ^x^	71.8 ^y^	2.567	0.062
Breast muscle, %	20.1	19.7	1.038	0.293
Heart, %	0.44	0.42	0.057	0.149
Gizzard, %	0.79	0.82	0.092	0.184
Liver, %	1.95	2.01	0.160	0.402

* Sixteen replicates/birds per treatment; T1—Maxiban^®^/Monteban^®^; T2—Maxiban^®^/Sacox^®^; BWbs = body weight before slaughter = 100%, CW = carcass weight, DP = dressing percentage. Values in the same row with no common superscript (a,b) are significantly different (*p* < 0.05), and 0.05 ≤ *p* < 0.10 (x,y) is considered a near-significant trend.

**Table 8:** Lesion scores of challenged chickens on days 21 and 28.

			Lesion Score ^1^			
*Eimeria* Strain	Day of HTSi	Treatment ^2^	0	1	2	3
*E*. *acervulina*	Day 21	T1	14	2	0	0
		T2	15	1	0	0
	Day 28	T1	16	0	0	0
		T2	14	2	0	0
*E*. *maxima*	Day 21	T1	5	11	0	0
		T2	10	6	0	0
	Day 28	T1	8	7	0	1
		T2	13	2	0	1
*E*. *tenella*	Day 21	T1	16	0	0	0
		T2	16	0	0	0
	Day 28	T1	16	0	0	0
		T2	16	0	0	0

Sixteen replicates/birds per treatment, for lesion scores by randomly selecting one bird per pen, which were examined on day 21 and day 28. T1—Maxiban^®^/Monteban^®^; T2—Maxiban^®^/Sacox^®^, HTSi—health tracking system. ^1^ Lesion score, 0 = gross lesions absent, 1 = few scattered lesions, 2 = greater number of discrete lesions involving more of the affected zone of the intestine and marked bleeding with *Eimeria*, 3 = higher number of lesions. ^2^ The broilers were challenged into the crop with an Evant vaccine (40 times the recommended dose) on days 14 and 21 of the trial. The coccidia vaccine contained *Eimeria acervulina* (003), *Eimeria maxima* (013), *Eimeria mitis* (006), *Eimeria praecox* (007), and *Eimeria tenella* (004) strains of *Eimeria*.

## Data Availability

The datasets generated and/or analyzed during the current study are available from the corresponding author upon reasonable request.
